# Plasma Biomarkers for Clinical Assessment of Bone Mineral Density in Heart Transplanted Patients—A Single-Center Study at Skåne University Hospital in Lund

**DOI:** 10.3389/ti.2022.10161

**Published:** 2022-03-28

**Authors:** Eveline Löfdahl, Salaheldin Ahmed, Abdulla Ahmed, Göran Rådegran

**Affiliations:** ^1^ Cardiology, Department of Clinical Sciences Lund, Lund University, Lund, Sweden; ^2^ The Section for Heart Failure and Valvular Disease, VO. Heart and Lung Medicine, Skåne University Hospital, Lund, Sweden

**Keywords:** heart transplantation, plasma biomarkers, bone mineral density, osteoporosis, bone metabolism

## Abstract

We aimed to identify plasma biomarkers that predict changes in bone mineral density (BMD) and increase the understanding of impaired BMD after heart transplantation (HT). Twenty-eight adult patients were included. Data, including densitometry and 29 plasma proteins, before and 1 year after HT were analyzed. Pre-HT plasma levels of fibroblast growth factor 23 (FGF23) correlated with post-HT T score in lumbar spine, adjusted for age, gender, and BMI (1.72 [95% CI 1.33; 2.22], *p* = 0.011). Change (∆; post-HT—pre-HT) in plasma levels of melusin correlated to ∆T score from the lumbar spine (*p* = 0.028). ∆plasma levels of TR-AP, ITGB2, and Stromelysin-1 correlated to ∆T score from the femoral neck (*p* < 0.05). However, no correlations remained after adjustments for age, gender, and BMI. In conclusion, elevated plasma FGF23 pre-HT predicted an increase in lumbar BMD after HT. However, the results are surprising since FGF23 is known to be inversely correlated with BMD. This may partly be explained by the complex pathophysiology in this particular cohort. Due to the explorative nature of the study and the small sample size, further investigations of biochemical markers on bone metabolism in this patient population are encouraged.

## Graphical Abstract

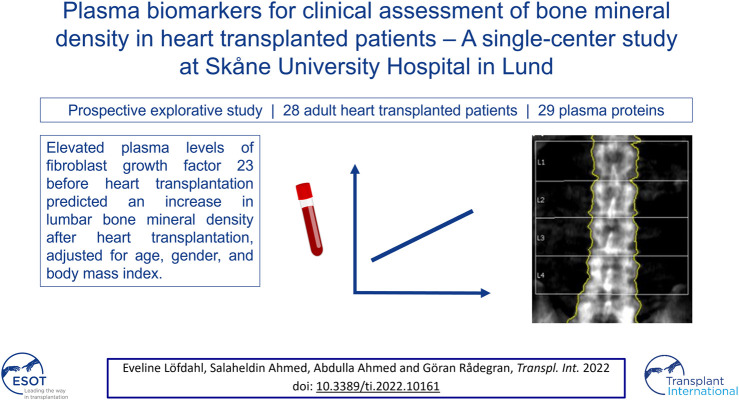

## Introduction

Osteoporosis is a common condition in patients who have undergone heart transplantation (HT) [1]. It may arise as a side effect of the immunosuppressive therapy given after HT, or as a consequence of various factors related to the heart failure prior to HT, including immobilization, impaired renal function, and heart failure medications [2-7]. Osteoporosis increases the risk of bone fractures which increase morbidity and mortality rates, of which the excess mortality rate within the first year after a hip fracture has been found to range from 8.4% to 36% [8-10]. Also, about 50% of patients who suffer a hip fracture are not able to walk independently afterwards long-term [10]. It has previously been reported that the mortality rate increases 1.5-fold for each standard deviation (SD) decrease in bone mineral density (BMD) in patients with osteoporosis [11]. Hence, impaired bone health constitutes a major limitation for survival and quality of life after HT. Early identification and treatment of osteoporosis are therefore of great clinical interest.

Emerging indicators of bone disease are biochemical markers which reflect the dynamics of bone metabolism, i.e., the process of bone formation and bone resorption [12]. Markers for bone formation reflect the function and recruitment of osteoblasts, including alkaline phosphatase (total and bone-specific), osteocalcin, and procollagen type I N-terminal propeptide, which all can be measured in plasma [13]. Markers for bone resorption, on the other hand, reflect the byproducts of osteoclast activity and include hydroxyproline, pyridinoline, and deoxypyridinoline, which are found in urine, whereas N-terminal and C-terminal crosslinked peptides can be found in both plasma and urine [13].

The current gold standard for assessment of bone strength is BMD which is measured using Dual-energy X-ray absorptiometry (DXA) [8]. Although DXA is widely available and provides a non-invasive method of bone strength assessment, it is also considered a static measurement of bone strength and a relatively expensive investigation, with a reported median cost of $98 per investigation in the Unites States in 2010 [13, 14]. It has been hypothesized that biochemical markers of bone metabolism may prove to be more useful than DXA as they are non-invasive, relatively inexpensive, and due to increasing availability of clinical chemistry analyzers in laboratories [15].

Whether biochemical biomarkers on bone metabolism before HT are useful in assessing bone health after HT is, however, unclear. Therefore, we aimed to identify plasma biomarkers that may predict changes in BMD and increase the understanding of impaired BMD after HT.

## Patients and Methods

### Study Design and Patient Selection

In the present observational cohort study, 29 patients with advanced heart failure were enrolled between October 2011 and July 2015. Patients were evaluated before and 1-year after HT, during the routine clinical evaluations at Skåne University Hospital, Lund, Sweden. Inclusion criteria were adult patients (≥18 years old) available in Lund Cardio Pulmonary Registry (LCPR), a prospective cohort of blood samples and clinical data, and a part of Region Skåne Biobank. Blood samples were collected at the time of inclusion and at the 1-year follow-up. Diagnostic and transplantation procedures were conducted at Skåne University hospital in Lund, Sweden, in accordance with the prevailing guidelines of The International Society for Heart and Lung Transplantation at the time of inclusion [16, 17].

Written informed consents were acquired from all patients upon enrollment. The study was approved by the local ethical board in Lund, Sweden (diary numbers: 2010/114; 2010/442; 2011/368; 2011/777; 2014/92 and 2015/270) and was conducted in agreement with the declarations of Helsinki and Istanbul.

### Blood Sampling and Protein Analysis

Between October 2011 and February 2017, venous, non-fasting, blood samples were collected in ethylenediaminetetraacetic acid (EDTA) vacutainer tubes from patients during the routine clinical evaluations before- and at the 1-year follow-up after HT. The blood samples were thereafter centrifuged at 2,000 rpm × 10 min at 20°C and plasma aliquots subsequently stored in LCPR at −80°C.

Twenty-nine proteins related to bone metabolism were analysed in May 2017 using the following multiplex immunoassay panels (Proseek Multiplex cardiovascular II, cardiovascular III and Oncology II panels, Olink Proteomics, Uppsala, Sweden). Proximity extension assay is based on protein specific oligonucleotide-linked antibodies and quantitative microfluidic PCR for protein detection. When a pair of antibodies are in proximity due to binding to the target protein, their respective oligonucleotide strands hybridize, forming a protein-unique DNA reporter sequence, which is subsequently used to quantify the proteins using real-time PCR [18].

The twenty-nine proteins analysed were cadherin-5, CCN family member 1 (CCN1), collagen alpha-1(I) chain (COL1A1), decorin, fibroblast growth factor 23 (FGF23), glypican-1, integrin alpha-V (ITGAV), integrin beta-2 (ITGB2), integrin beta-5 (ITGB5), matrilysin, matrix extracellular phosphoglycoprotein (MEPE), matrix metalloproteinase (MMP)2, MMP9, MMP12, melusin, metalloproteinase inhibitor 4 (TIMP4), osteoclast-associated immunoglobulin-like receptor (hOSCAR), osteonectin, osteopontin, osteoprotegerin, perlecan, prolargin, receptor activator of nuclear factor κ-B (RANK), stromelysin-1, syndecan-1, tartrate-resistant acid phosphatase type 5 (TR-AP), thrombospondin-2, transmembrane glycoprotein NMB (GPNMB), and WNT1-inducible-signaling pathway protein 1 (WISP1).

The proteins’ levels were expressed in a log2 normalized protein expression scale (NPX) as arbitrary units, corresponding to the inverted Ct-values, unless otherwise stated, i.e., linear NPX [18]. All panels are validated regarding sensitivity, dynamic range, specificity, precision, and scalability. Information about panel specific validation can be found at www.olink.com/downloads.

### Bone Mineral Density and Other Data Collection

Measurements of BMD was collected from clinical records during the transplantation assessment before HT and from the routine check-up 1 year after HT. BMD was expressed in T score (SD) and was obtained using DXA from the lumbar spine and femoral neck.

Other data collected included age (recipient), gender, body mass index (BMI [kg/m^2^]), primary indications for HT, and administration of systemic corticosteroids (CS). Glomerular filtration rate (GFR [ml/min/1.73 m^2^]) was based on measurement of iohexol clearance or serum levels of creatinine (i.e., estimated [e]GFR). The eGFR was calculated using the CKD-EPI formula, in accordance with the current guidelines from the Kidney Disease: Improving Global Outcomes (KDIGO) working formulation [19].

### Study Setup

To explore the predictive value of protein levels and BMD, correlations between pre-HT protein levels and post-HT T score in lumbar spine and femoral neck were performed. Next, to reflect the dynamics of protein levels in relation to the dynamics of BMD, correlations of Δ (delta; post-HT—pre-HT values) protein levels vs. ΔT score in lumbar spine and femoral neck were performed.

### Statistical Analysis

Linear regression models were employed to describe the relation between each of the plasma protein levels pre-HT and T score from the lumbar spine and femoral neck post-HT, respectively. Similarly, the relation between Δplasma protein levels and ΔT score from the lumbar spine and femoral neck was investigated in linear regression models. We adjusted for multiple testing using the Benjamini and Hochberg (false discovery rate) correction (Q = 5%). We used Pearson correlation coefficients to evaluate the relationship between pre-HT plasma protein levels and post-HT T scores as well as the relationship between Δplasma protein levels and ΔT scores. Simple linear regressions were calculated in order to predict pre-HT plasma levels of FGF23 by GFR based on iohexol clearance and serum levels of creatinine. All analyses were performed in R v.4.1 (R Core Development Team 2021), and a *p*-value of <0.05 was considered statistically significant. The median and interquartile range (IQR) were calculated for continuous variables.

## Results

### Study Population

Of the 29 patients, one was retransplanted and was therefore excluded. Of the remaining included 28, pre-HT data was collected at a median of 115 (70; 237) days before HT and post-HT data was collected at a median of 395 (369; 429) days after HT. The most frequent primary indication for HT was dilated cardiomyopathy (68%). Baseline characteristics, as well as follow-up data 1 year after HT, are displayed in Table 1.

**TABLE 1:** Patient characteristics.

Recipient characteristics	Pre-HT			Post-HT		
	*N* = 28		Missing	*N* = 28		Missing
Age (years)	50	(45; 60)	0	52	(47; 62)	0
Female, *N* (%)	6	(21)	0	6	(21)	0
BMI (kg/m^2^)	27	(24; 28)	1	26	(23; 30)	0
Serum creatinine (µmol/L)	106	(88; 121)	0	114	(97; 142)	0
Creatinine based eGFR	65	(57; 82)	0	54	(45; 75)	0
Iohexol-GFR (ml/min/1.73 m^2^)	56	(45; 69)	13	53	(46; 78)	2
Daily administration of systemic CS, *N* (%)	1	(4)	0	27	(96)	0
Primary indication for HT			0			
Dilated cardiomyopathy	19	(68)				
Hypertrophic cardiomyopathy	2	(7)				
Ischemic cardiomyopathy	2	(7)				
Other	5	(18)				
BMD (g/m^2^)						
Lumbar spine	1.135	(1.028; 1.272)		1.113	(0.944; 1.188)	3
Femoral neck	1.001	(0.946; 1.063)		0.904	(0.818; 0.966)	3
T score (SD)						
Lumbar spine	−0.7	(−1.6; 0.4)		−1.0	(−2.3; −0.2)	2
Femoral neck	−0.7	(−1.0; −0.1)		−1.4	(−1.9; −0.9)	2

Values for continuous variables are expressed as median (IQR), whereas categorical values are expressed as number (%). BMD, bone mineral density; BMI, body mass index; CS, corticosteroids; HT, heart transplantation; (e)GFR, (estimated) glomerular filtration rate; IQR, interquartile range; SD, standard deviation.

### Maintenance Immunosuppressive Therapy

Immunosuppressive agents were tapered after HT in accordance with local guidelines, previously described elsewhere [20]. A total of 64% received a combination of prednisolone, tacrolimus, and mycophenolate mofetil; 14% received prednisolone, cyclosporine, and mycophenolate mofetil; 14% received prednisolone, tacrolimus, and azathioprine; whereas 7% received other combinations. Only one patient was completely free of systemic corticosteroids at the 1-year post-HT check-up.

### Pre-HT FGF23 Correlated Independently With Post-HT T Score in the Lumbar Spine

**FIGURE 1:**
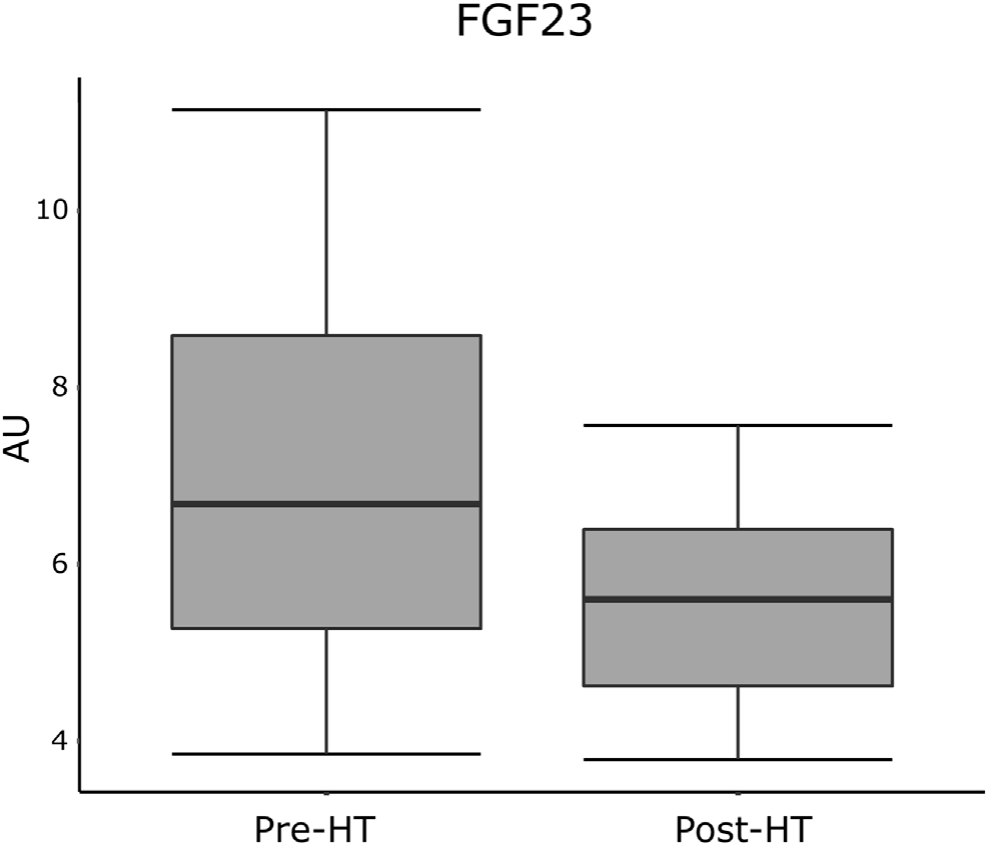
Plasma levels of FGF23 from pre- and post-HT measured in normalized protein expression scale, expressed in AU. AU, arbitrary units; FGF23, fibroblast growth factor 23; HT, heart transplantation.

In linear regression analyses, pre-HT plasma levels of FGF23 correlated with post-HT T score in lumbar spine adjusted for age, gender, and BMI (1.72 [95% CI 1.33; 2.22], *p* = 0.011). All correlations between pre-HT levels of proteins and post-HT T score from the lumbar spine and femoral neck are presented in Table 2. Protein levels from both pre- and post-HT are displayed in boxplots in Figure 1 (FGF23) and in Supplementary Figure S1 (remainder). Correlations between pre-HT plasma protein levels and post-HT T score from the lumbar spine and femoral neck are shown in Supplementary Figure S2.

**TABLE 2:** Regression analyses between pre-HT levels of plasma proteins measured in normalized protein expression scale, expressed in AU, and post-HT T score from the lumbar spine (A) and femoral neck (B). ^a^Adjusted with Benjamini & Hochberg (false discovery rate) correction.

(A) Plasma protein	*β*	(95% CI)	*p*	Adjusted *p* ^a^
FGF23	1.72	(1.33; 2.22)	<0.001*	0.011*
Osteopontin	2.44	(1.40; 4.27)	0.005*	0.066
Osteoprotegerin	3.21	(1.30; 7.91)	0.018*	0.111
Perlecan	2.24	(1.22; 4.11)	0.016*	0.111
RANK	2.02	(1.17; 3.49)	0.019*	0.111
COL1A1	2.20	(1.14; 4.27)	0.028*	0.135
ITGB2	2.18	(1.03; 4.63)	0.053	0.219
Melusin	1.22	(1.00; 1.50)	0.066	0.240
WISP1	1.78	(0.94; 3.36)	0.087	0.258
ITGB5	2.32	(0.92; 5.88)	0.089	0.258
Stromelysin-1	1.51	(0.93; 2.43)	0.107	0.259
MEPE	1.79	(0.91; 3.54)	0.106	0.259
MMP2	2.59	(0.80; 8.42)	0.126	0.261
MMP9	1.52	(0.92; 2.51)	0.117	0.261
Prolargin	3.43	(0.64; 18.40)	0.164	0.317
Syndecan-1	1.71	(0.79; 3.70)	0.184	0.334
Matrilysin	1.39	(0.78; 2.47)	0.270	0.459
TIMP4	1.48	(0.73; 2.99)	0.285	0.459
Osteonectin	2.10	(0.46; 9.58)	0.349	0.533
Glypican-1	1.60	(0.54; 4.72)	0.403	0.584
ITGAV	1.85	(0.35; 9.80)	0.478	0.660
Cadherin-5	1.38	(0.47; 4.07)	0.566	0.746
Decorin	1.25	(0.50; 3.13)	0.631	0.766
HOSCAR	1.52	(0.28; 8.32)	0.634	0.766
TR-AP	0.83	(0.32; 2.16)	0.703	0.778
Thrombospondin-2	0.79	(0.22; 2.90)	0.724	0.778
MMP12	1.08	(0.76; 1.54)	0.681	0.778
GPNMB	1.11	(0.16; 7.57)	0.917	0.950
CCN1	1.00	(0.41; 2.44)	0.994	0.994
**(B) Plasma protein**				
Melusin	1.15	(1.02; 1.28)	0.029*	0.758
GPNMB	0.40	(0.14; 1.12)	0.095	0.758
HOSCAR	0.44	(0.18; 1.12)	0.100	0.758
ITGB5	1.54	(0.90; 2.66)	0.130	0.758
Osteopontin	1.33	(0.92; 1.92)	0.138	0.758
COL1A1	1.35	(0.90; 2.03)	0.159	0.758
Thrombospondin-2	0.60	(0.29; 1.24)	0.183	0.758
FGF23	1.10	(0.91; 1.33)	0.313	0.947
Syndecan-1	0.82	(0.52; 1.29)	0.405	0.947
MMP12	0.92	(0.75; 1.13)	0.436	0.947
ITGB2	1.19	(0.75; 1.90)	0.465	0.947
TR-AP	0.82	(0.47; 1.41)	0.478	0.947
Prolargin	0.72	(0.26; 1.96)	0.525	0.947
Osteonectin	1.33	(0.55; 3.23)	0.531	0.947
Stromelysin-1	1.08	(0.81; 1.44)	0.618	0.947
Cadherin-5	0.86	(0.46; 1.61)	0.642	0.947
MMP9	1.07	(0.79; 1.45)	0.682	0.947
MEPE	0.92	(0.61; 1.39)	0.696	0.947
CCN1	1.11	(0.66; 1.85)	0.704	0.947
Perlecan	0.93	(0.63; 1.38)	0.732	0.947
Osteoprotegerin	1.10	(0.61; 1.97)	0.760	0.947
ITGAV	1.16	(0.44; 3.06)	0.767	0.947
Matrilysin	0.96	(0.68; 1.34)	0.799	0.947
Glypican-1	0.93	(0.49; 1.75)	0.816	0.947
RANK	1.04	(0.73; 1.49)	0.819	0.947
Decorin	1.04	(0.62; 1.77)	0.874	0.947
WISP1	1.02	(0.69; 1.51)	0.913	0.947
MMP2	0.97	(0.48; 1.99)	0.941	0.947
TIMP4	0.99	(0.65; 1.49)	0.947	0.947

a Adjusted with Benjamini & Hochberg (false discovery rate) correction.

AU, arbitrary units; CCN1, CCN family member 1; CI, confidence interval; COL1A1, collagen alpha-1(I) chain; FGF23, fibroblast growth factor 23; ITGAV, integrin alpha-V; ITGB2, integrin beta-2; ITGB5, integrin beta-5; MEPE, matrix extracellular phosphoglycoprotein; MMP, matrix metalloproteinase; TIMP4, metalloproteinase inhibitor 4; hOSCAR, osteoclast-associated immunoglobulin-like receptor; RANK, receptor activator of nuclear factor κ-B; TR-AP, tartrate-resistant acid phosphatase type 5; GPNMB, transmembrane glycoprotein NMB; WISP1, WNT1-inducible-signaling pathway protein 1. *Indicates statistical significance.

In a sub -analysis, pre-HT plasma levels of FGF23 were inversely correlated with pre-HT iohexol based GFR, suggesting a FGF23 factor decrease of 0.99 (95% CI 0.97; 1.00) arbitrary units (AU) (*p* = 0.029). Likewise, pre-HT FGF23 levels decreased with a factor of 0.99 (95% CI 0.97; 1.00) AU by every unit increase in pre-HT creatinine-based GFR, however, this relationship was statistically not significant (*p* = 0.072).

### Dynamics of Plasma Protein Levels in Relation to Bone Mineral Density Evolution

Regression analyses between Δplasma protein levels and ∆T score from the lumbar spine and femoral neck are shown in Table 3. In the unadjusted analysis, ∆plasma levels of melusin correlated to ∆T score from the lumbar spine (1.20 [95% CI 1.03; 1.40], *p* = 0.028). ∆plasma levels of TR-AP, ITGB2, and Stromelysin-1 correlated to ∆T score from the femoral neck (1.23 [95% CI 1.07; 1.42], 1.25 [95% CI 1.03; 1.52], and 0.90 [95% CI 0.81; 0.99], respectively, all with *p* < 0.05). However, after adjustments for age, gender, and BMI, no significant correlations remained. Correlations between Δplasma protein levels and ∆T score from the lumbar spine and femoral neck are shown in Supplementary Figure S3.

**TABLE 3:** Regression analyses between Δplasma protein levels measured in normalized protein expression scale, expressed in AU, and ∆T score from the lumbar spine (A) and femoral neck (B).

(A) Plasma protein	*β*	(95% CI)	*p*	Adjusted *p* ^a^
ΔMelusin	1.20	(1.03; 1.40)	0.028*	0.809
ΔOsteoprotegerin	1.01	(0.48; 2.14)	0.974	0.993
ΔCCN1	0.96	(0.48; 1.94)	0.912	0.993
ΔWISP1	1.02	(0.62; 1.68)	0.935	0.993
ΔCOL1A1	1.34	(0.75; 2.39)	0.337	0.993
ΔITGB2	0.84	(0.42; 1.69)	0.624	0.993
ΔITGAV	0.53	(0.15; 1.88)	0.334	0.993
ΔDecorin	0.58	(0.13; 2.61)	0.485	0.993
ΔMMP2	1.04	(0.54; 2.02)	0.908	0.993
ΔStromelysin-1	0.97	(0.67; 1.40)	0.868	0.993
ΔMatrilysin	0.81	(0.38; 1.72)	0.589	0.993
ΔOsteonectin	3.25	(0.55; 19.20)	0.206	0.993
ΔOsteopontin	1.25	(0.86; 1.81)	0.259	0.993
ΔTR-AP	1.14	(0.67; 1.94)	0.641	0.993
ΔMMP9	1.31	(0.97; 1.77)	0.089	0.993
ΔITGB5	1.55	(0.55; 4.35)	0.417	0.993
ΔSyndecan-1	1.23	(0.84; 1.81)	0.298	0.993
ΔCadherin-5	0.34	(0.09; 1.25)	0.117	0.993
ΔGlypican-1	0.84	(0.31; 2.25)	0.725	0.993
ΔThrombospondin-2	0.77	(0.29; 2.07)	0.615	0.993
ΔMMP12	1.02	(0.65; 1.62)	0.926	0.993
ΔProlargin	0.78	(0.22; 2.79)	0.706	0.993
ΔPerlecan	1.27	(0.49; 3.25)	0.628	0.993
ΔGPNMB	0.49	(0.06; 3.97)	0.512	0.993
ΔhOSCAR	0.88	(0.19; 4.17)	0.877	0.993
ΔTIMP4	1.02	(0.59; 1.77)	0.934	0.993
ΔFGF23	1.04	(0.88; 1.22)	0.669	0.993
ΔMEPE	1.00	(0.42; 2.39)	0.993	0.993
ΔRANK	1.47	(0.86; 2.49)	0.169	0.993
**(B) Plasma protein**				
ΔTR-AP	1.23	(1.07; 1.42)	0.007*	0.189
ΔITGB2	1.25	(1.03; 1.52)	0.032*	0.435
ΔStromelysin-1	0.90	(0.81; 0.99)	0.045*	0.435
ΔMMP2	0.83	(0.69; 1.00)	0.063	0.457
ΔGPNMB	0.60	(0.33; 1.06)	0.094	0.481
ΔTIMP4	0.88	(0.75; 1.03)	0.116	0.481
ΔFGF23	0.96	(0.92; 1.01)	0.106	0.481
ΔOsteonectin	1.47	(0.88; 2.46)	0.158	0.528
ΔMelusin	1.04	(0.99; 1.09)	0.164	0.528
ΔProlargin	0.77	(0.53; 1.12)	0.184	0.534
ΔCCN1	1.14	(0.93; 1.39)	0.213	0.547
ΔCOL1A1	0.90	(0.76; 1.07)	0.238	0.547
ΔITGB5	1.20	(0.89; 1.62)	0.245	0.547
ΔWISP1	0.92	(0.80; 1.06)	0.280	0.557
ΔDecorin	1.28	(0.82; 2.01)	0.288	0.557
ΔITGAV	0.83	(0.57; 1.21)	0.341	0.618
ΔThrombospondin-2	0.88	(0.65; 1.18)	0.387	0.624
ΔPerlecan	0.88	(0.66; 1.17)	0.381	0.624
ΔOsteopontin	1.04	(0.93; 1.17)	0.471	0.719
ΔMatrilysin	0.94	(0.75; 1.17)	0.569	0.745
ΔGlypican-1	0.92	(0.69; 1.23)	0.575	0.745
ΔMMP12	1.04	(0.91; 1.19)	0.591	0.745
ΔRANK	1.05	(0.89; 1.24)	0.564	0.745
ΔOsteoprotegerin	0.95	(0.75; 1.18)	0.627	0.758
ΔMEPE	0.94	(0.73; 1.22)	0.658	0.763
ΔSyndecan-1	0.98	(0.87; 1.10)	0.714	0.796
ΔCadherin-5	0.95	(0.63; 1.43)	0.801	0.830
ΔhOSCAR	1.07	(0.67; 1.71)	0.791	0.830
ΔMMP9	1.01	(0.91; 1.11)	0.910	0.910

a Adjusted with Benjamini & Hochberg (false discovery rate) correction.

Δ, delta (post-HT—pre-HT values); AU, arbitrary units; CCN1, CCN family member 1; CI, confidence interval; COL1A1, collagen alpha-1(I) chain; FGF23, fibroblast growth factor 23; ITGAV, integrin alpha-V; ITGB2, integrin beta-2; ITGB5, integrin beta-5; MEPE, matrix extracellular phosphoglycoprotein; MMP, matrix metalloproteinase; TIMP4, metalloproteinase inhibitor 4; hOSCAR, osteoclast-associated immunoglobulin-like receptor; RANK, receptor activator of nuclear factor κ-B; TR-AP, tartrate-resistant acid phosphatase type 5; GPNMB, transmembrane glycoprotein NMB; WISP1, WNT1-inducible-signaling pathway protein 1. *Indicates statistical significance.

## Discussion

Impaired BMD is commonly found in patients who have undergone HT, leading to significant impact on morbidity and mortality [9, 10]. Early risk stratification and prevention of the development of osteoporosis is therefore of great interest. Emerging indicators of bone disease are plasma bone turnover markers which reflect the dynamics of bone metabolism. Such biochemical markers are considered beneficial with regard to availability and cost-effectiveness when compared to DXA which constitutes the current gold standard method for assessment of BMD [15]. Hence, identification of biochemical markers for the prediction of osteoporosis after HT is of particular interest.

The present single-center observational cohort study aimed to identify plasma biomarkers that may predict changes in BMD and increase the understanding of impaired BMD after HT. This may enable better prediction of impaired skeletal health and improve outcome in this patient population. The present study showed that plasma levels of FGF23 before HT correlated with T score in the lumbar spine after HT, independent of age, gender, and BMI. However, no correlations between changes in plasma levels of biochemical markers and T scores were found. The findings suggest that post-HT BMD loss may be predicted by pre-HT measurements of serum FGF23.

A positive correlation was found between pre-HT levels of FGF23 and post-HT T score in lumbar spine. FGF23, mainly secreted by osteocytes and osteoblasts in bone, plays a significant role in bone mineralization by stimulating phosphaturia, as well as suppressing the production of 1,25-dihydroxyvitamin D, resulting in inhibited bone mineralization [21]. Thus, the findings of the present study are contradictory. In a study by Valentin et al. (2013) on mutant mice, it was concluded that FGF23 plasma levels strongly correlates with circulating calcium levels, suggesting that suppressed FGF23 levels protects from hypocalcemia by reduced inhibitory effect on 1,25-dihydroxyvitamin D production [22]. To our knowledge, no previous study on the correlation of FGF23 levels and BMD after HT have been conducted. However, Jovanovich et al. (2013) found in a prospective, longitudinal study of community-dwelling adults aged 65 or older, including >3,000 participants with a median follow-up of 9.6 (IQR = 5.1; 11.0) years, that FGF23 was weakly associated with increased BMD in both lumbar spine and hip, but no associations were detected between FGF23 levels and fracture risk [23]. Similarly, FGF23 correlated positively with BMD in lumbar spine and hip in a study by Marsell et al. (2008), including >3,000 male participants aged 69–80 years [24]. However, the correlations were discovered to be dependent on BMI. Thus, these results partly support those of the present study. In addition, FGF23 levels are also known to increase in relation to progression of kidney dysfunction, which is common in HT candidates [25]. In a previous study at our center, it was concluded that the occurrence of kidney dysfunction, measured by iohexol clearance, increased over time after HT, reaching 25% with CKD stage ≥4 by the fifth post-operative year [26]. It is furthermore known that DXA from the lumbar spine might be overestimated in cases of vascular calcification, which is a common feature in patients with chronic kidney disease [27, 28]. All in all, FGF23 predicted a higher lumbar T score after HT, which may partly be explained by the complex pathophysiological mechanisms in this particular patient cohort.

Plasma levels of FGF23 correlated positively with T score in the lumbar spine, but not with T score in the femoral neck. In a cross-sectional study, Rupp et al. assessed levels of FGF23 and bone microarchitecture in 82 patients with osteoporosis [29]. They concluded that increased levels of FGF23 were associated with impaired trabecular but not cortical bone microarchitecture, after adjusting for age and BMI. This is contradicting to our results, but may be partly explained by overestimations of T score in the lumbar spine, as outlined above, as well as the potential impact of renal dysfunction as pre-HT levels of FGF23 correlated with both measured and estimated GFR pre-HT.

A correlation between the change from pre-HT to post-HT in plasma levels of melusin, a muscle-specific integrin beta1-interacting protein, and the change in lumbar T score was found in the unadjusted analysis. However, no correlation remained after adjustments for age, gender, and BMI. It is well known that beta1 integrins are required for proper bone formation and homeostasis by playing a main role in the recruitment, differentiation, and mineralization of osteoblasts [30-32]. Brunner et al. (2018) reported that, for proper bone formation, beta1 integrins are required at the early stages of osteoblast differentiation *in vivo* [33]. Thus, the findings of the present study may reflect the pathophysiology behind beta1 integrins and their impact on bone formation.

Further, in the unadjusted analysis, the change from pre-HT to post-HT in plasma levels of ITGB2, stromelysin-1, and TR-AP correlated with the change in femoral T score. After adjustments for age, gender, and BMI, however, these correlations were lost. Although TR-AP has been considered a marker for osteoclastic activity, Halling Linder et al. (2017) demonstrated that TR-AP exhibits an inhibitory effect on osteopontin mediated mineralization delay, which is supported by the findings of this study [34, 35]. Also, ITGB2, which is involved in cell adhesion and in promoting intracellular signaling events, has been found to play a key role in the osteogenic processes [36, 37]. Miura et al. (2005) showed that mice lacking CD18, one of the members in beta-2 integrin family, exhibited features of osteoporosis, including decreased BMD, and impaired trabecular microarchitecture. This is consistent with the positive correlation between ∆plasma levels of ITGB2 and ∆T score in the femoral neck that was found in the present study [37]. Stromelysin-1 is an activator of procollagenases which promotes cartilage degeneration [38]. In a study by Blom et al. (2007), stromelysin-1-knockout mice demonstrated a significant reduction in cartilage degeneration after induction of osteoarthritis [39]. Whether stromelysin-1 has an impact on the development of osteoporosis in HT patients remains to be established.

The present study provides explorative data on novel biochemical plasma markers for bone metabolism in 28 patients after HT. The major strength of this study was the application of multiplex proximity extension assay, which is known for its high sensitivity and specificity in plasma [18]. Data was independent of fasting and was adjusted for age, gender, and BMI. Moreover, the study was performed at a single-center which facilitated data collection. Due to the explorative nature of the study, the small size of the patient cohort, as well as absence of a validation cohort, generalizability of the results is limited. Furthermore, the small size of the study restricted statistical adjustments with additional variables, such as comorbidities, medications, CS dose, time on waiting list, vitamin D intake and serum level, as well as calcium and phosphate levels in serum and urine, potentially influencing the BMD and levels of biochemical markers.

In conclusion, the present study showed that elevated plasma levels of FGF23 pre-HT predicted an increase in lumbar BMD after HT, which may be partly explained by the complex pathophysiological mechanisms in relation to the comorbid burden and immunosuppressive therapy in this patient cohort. Further investigations of biochemical markers on bone metabolism, especially FGF23, in larger HT populations are highly encouraged.

## Data Availability

The raw data supporting the conclusion of this article will be made available by the authors, without undue reservation.

## Supplementary Material

Supplementary Figure 1 Plasma levels of proteins from pre- and post-HT measured in normalized protein expression scale, expressed in AU. AU, arbitrary units; CCN1, CCN family member 1; COL1A1, collagen alpha-1(I) chain; HT, heart transplantation; ITGAV, integrin alpha-V; ITGB2, integrin beta-2; ITGB5, integrin beta-5; MEPE, matrix extracellular phosphoglycoprotein; MMP, matrix metalloproteinase; TIMP4, metalloproteinase inhibitor 4; hOSCAR, osteoclast-associated immunoglobulin-like receptor; RANK, receptor activator of nuclear factor κ-B; TR-AP, tartrate-resistant acid phosphatase type 5; GPNMB, transmembrane glycoprotein NMB; WISP1, WNT1-inducible-signaling pathway protein 1.

Supplementary Figure 2
**(A-AC)** Correlations between pre-HT plasma protein levels measured in normalized protein expression scale, expressed in AU, and post-HT lumbar and femoral T scores. AU, arbitrary units; CCN1, CCN family member 1; COL1A1, collagen alpha-1(I) chain; FGF23, fibroblast growth factor-23; ITGAV, integrin alpha-V; ITGB2, integrin beta-2; ITGB5, integrin beta-5; MEPE, matrix extracellular phosphoglycoprotein; MMP, matrix metalloproteinase; TIMP4, metalloproteinase inhibitor 4; hOSCAR, osteoclast-associated immunoglobulin-like receptor; RANK, receptor activator of nuclear factor κ-B; TR-AP, tartrate-resistant acid phosphatase type 5; GPNMB, transmembrane glycoprotein NMB; WISP1, WNT1-inducible-signaling pathway protein 1.

Supplementary Figure 3 Correlations between Δplasma protein levels measured in normalized protein expression scale, expressed in AU, and ∆T score from the lumbar spine and femoral neck. AU, arbitrary units; CCN1, CCN family member 1; COL1A1, collagen alpha-1(I) chain; FGF23, fibroblast growth factor-23; ITGAV, integrin alpha-V; ITGB2, integrin beta-2; ITGB5, integrin beta-5; MEPE, matrix extracellular phosphoglycoprotein; MMP, matrix metalloproteinase; TIMP4, metalloproteinase inhibitor 4; hOSCAR, osteoclast-associated immunoglobulin-like receptor; RANK, receptor activator of nuclear factor κ-B; TR-AP, tartrate-resistant acid phosphatase type 5; GPNMB, transmembrane glycoprotein NMB; WISP1, WNT1-inducible-signaling pathway protein 1.

## Abbreviations

AU, arbitrary units; BMD, bone mineral density; BMI, body mass index; CCN1, CCN family member 1; CI, confidence interval; COL1A1, collagen alpha-1(I) chain; CS, corticosteroids; DXA, Dual-energy X-ray absorptiometry; EDTA, ethylenediaminetetraacetic acid; FGF23, fibroblast growth factor 23; GFR, glomerular filtration rate; GPNMB, transmembrane glycoprotein NMB; hOSCAR, osteoclast-associated immunoglobulin-like receptor; HT, heart transplantation; ITGAV, integrin alpha-V; ITGB2, integrin beta-2; ITGB5, integrin beta-5; IQR, interquartile range; LCPR, Lund Cardio Pulmonary Registry; MEPE, matrix extracellular phosphoglycoprotein; MMP2, matrix metalloproteinase-2; MMP9, matrix metalloproteinase-9; MMP12, macrophage metalloelastase; RANK, receptor activator of nuclear factor κ-B; SD, standard deviation; TIMP4, metalloproteinase inhibitor 4; TR-AP, tartrate-resistant acid phosphatase type 5; WISP-1, WNT1-inducible-signaling pathway protein 1.
