# Supplementary figures and images for: Plasma Biomarkers for Clinical Assessment of Bone Mineral Density in Heart Transplanted Patients—A Single-Center Study at Skåne University Hospital in Lund
Source: Transpl Int. 2022 Mar 28;35:10161. doi: 10.3389/ti.2022.10161 (PMC9487521; doi:10.3389/ti.2022.10161)

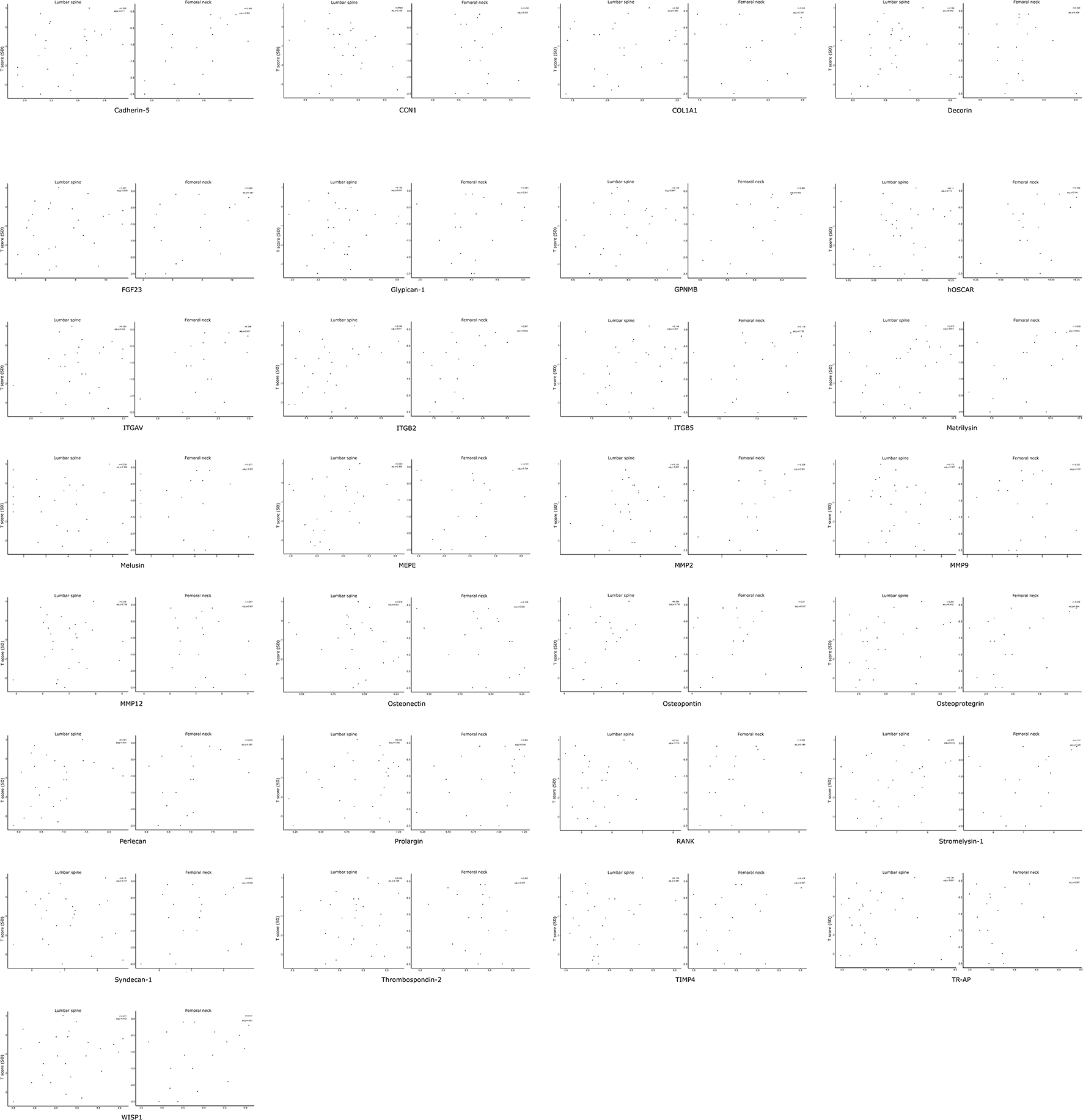

Supplement: Supplementary file 1 [file Image3.tif]

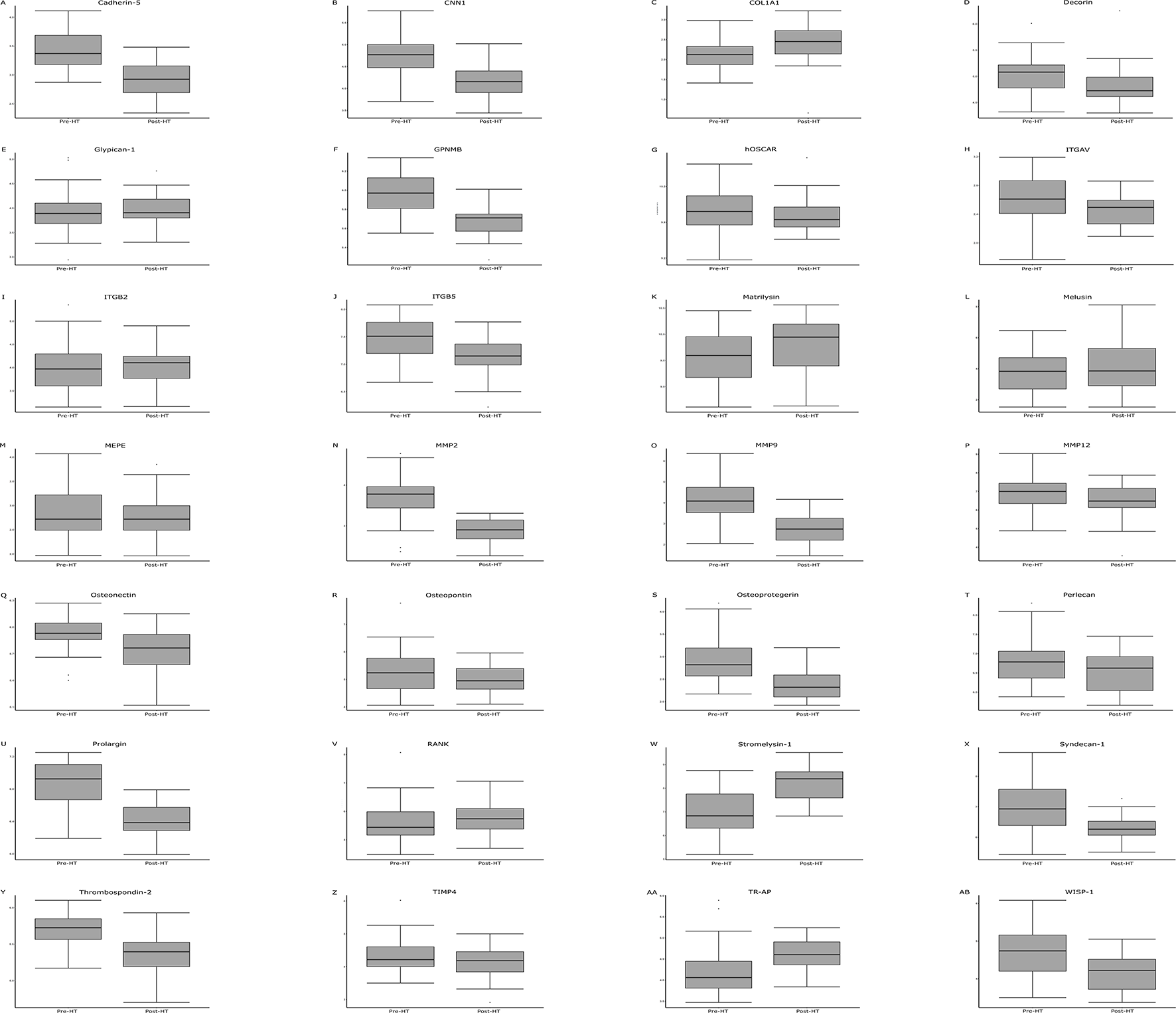

Supplement: Supplementary file 2 [file Image2.tif]

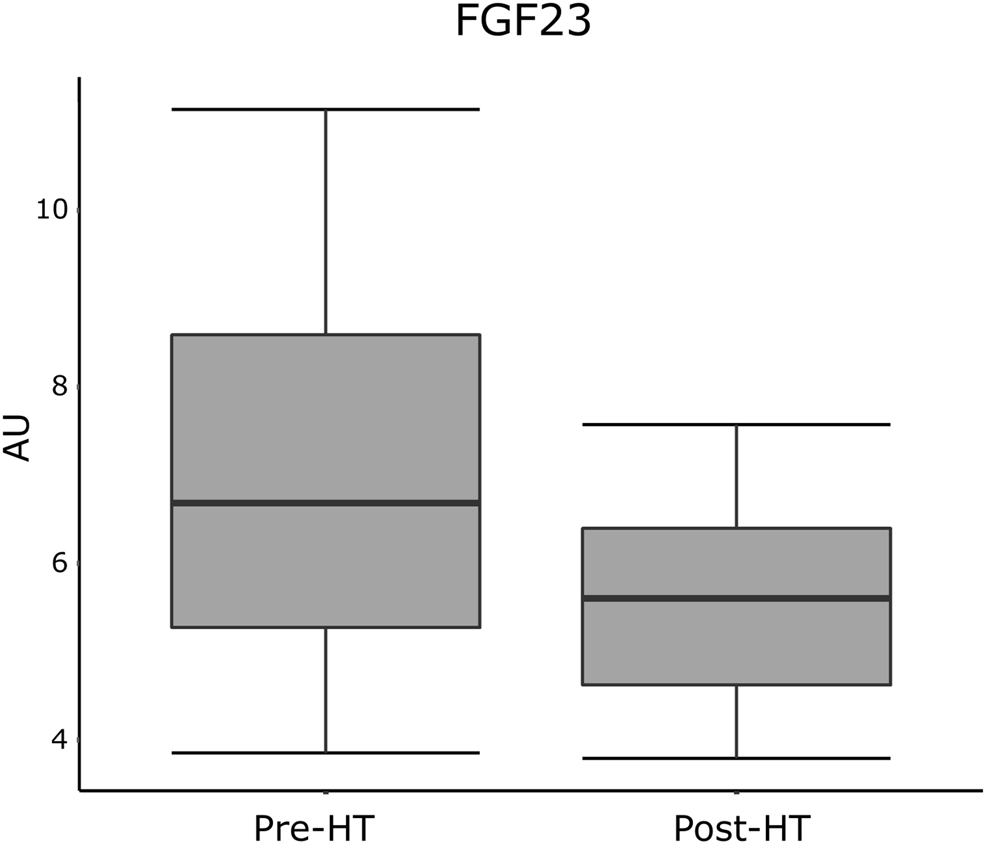

Supplement: Supplementary file 3 [file Image1.tif]
